# Validation of ICMR Neurocognitive Toolbox for Dementia in the Linguistically Diverse Context of India

**DOI:** 10.3389/fneur.2021.661269

**Published:** 2021-10-18

**Authors:** Mansi Verma, Manjari Tripathi, Ashima Nehra, Avanthi Paplikar, Feba Varghese, Suvarna Alladi, Jwala Narayanan, R. S. Dhaliwal, Meenakshi Sharma, Aralikatte Onkarappa Saroja, Faheem Arshad, Gollahalli Divyaraj, Amitabha Ghosh, Tejaswini S. Manae, Shailaja Mekala, Ramshekhar N. Menon, Roopa Hooda, Gowri K. Iyer, J. Sunitha, Rajmohan Kandukuri, Subhash Kaul, Arfa Banu Khan, Robert Mathew, Ranita Nandi, M. V. Padma, Apoorva Pauranik, Subasree Ramakrishnan, Lekha Sarath, Urvashi Shah, P. N. Sylaja, Ravi Prasad Varma, Yeshaswini Vishwanath

**Affiliations:** ^1^Department of Neurology, All India Institute of Medical Sciences, New Delhi, India; ^2^Clinical Neuropsychology, Neurosciences Centre, All India Institute of Medical Sciences, New Delhi, India; ^3^Department of Neurology, National Institute of Mental Health and Neurosciences, Bengaluru, India; ^4^Department of Neurology, Nizam's Institute of Medical Sciences, Hyderabad, India; ^5^Department of Neurology, Manipal Hospitals, Bengaluru, India; ^6^Indian Council of Medical Research, New Delhi, India; ^7^Department of Neurology, Jawaharlal Nehru Medical College, KLE Academy of Higher Education and Research, Belagavi, India; ^8^Cognitive Neurology Unit, Apollo Gleneagles Hospital, Kolkata, India; ^9^Department of Neurology, Sree Chitra Tirunal Institute for Medical Sciences and Technology, Thiruvananthapuram, India; ^10^Department of Psychiatry, KAHER's Jawaharlal Nehru Medical College and Research Center, Belagavi, India; ^11^Department of Neurology, Government Medical College, Alappuzha, India; ^12^Department of Neurology Mahatma Gandhi Mission Medical College, Indore, India; ^13^Department of Neurology, King Edward Memorial Hospital, Mumbai, India

**Keywords:** dementia, Alzheimer's disease, vascular dementia, neuropsychological assessment, cross cultural validation, India, ICMR-NCTB

## Abstract

**Objectives:** The growing prevalence of dementia, especially in low- and middle-income countries (LMICs), has raised the need for a unified cognitive screening tool that can aid its early detection. The linguistically and educationally diverse population in India contributes to challenges in diagnosis. The present study aimed to assess the validity and diagnostic accuracy of the Indian Council of Medical Research-Neurocognitive Toolbox (ICMR-NCTB), a comprehensive neuropsychological test battery adapted in five languages, for the diagnosis of dementia.

**Methods:** A multidisciplinary group of experts developed the ICMR-NCTB based on reviewing the existing tools and incorporation of culturally appropriate modifications. The finalized tests of the major cognitive domains of attention, executive functions, memory, language, and visuospatial skills were then adapted and translated into five Indian languages: Hindi, Bengali, Telugu, Kannada, and Malayalam. Three hundred fifty-four participants were recruited, including 222 controls and 132 dementia patients. The sensitivity and specificity of the adapted tests were established for the diagnosis of dementia.

**Results:** A significant difference in the mean (median) performance scores between healthy controls and patients with dementia was observed on all tests of ICMR-NCTB. The area under the curve for majority of the tests included in the ICMR-NCTB ranged from 0.73 to 1.00, and the sensitivity and specificity of the ICMR-NCTB tests ranged from 70 to 100% and 70.7 to 100%, respectively, to identify dementia across all five languages.

**Conclusions:** The ICMR-NCTB is a valid instrument to diagnose dementia across five Indian languages, with good diagnostic accuracy. The toolbox was effective in overcoming the challenge of linguistic diversity. The study has wide implications to address the problem of a high disease burden and low diagnostic rate of dementia in LMICs like India.

## Introduction

Dementia, a neurocognitive syndrome that affects the ability to perform everyday activities, has become a major health crisis worldwide, and research priorities that are aimed at reducing its global disease burden are a priority (1, 2). There has been a significant rise in the numbers of elderly people with dementia, especially in developing regions of the world (3–5). Of the 47 million people living with dementia globally, about 63% of these currently live in low- and middle-income countries (LMICs) (5–7). These figures are projected to further increase to 82 million by 2030 and 152 million by 2050, particularly in China, India, and Latin America (2, 8, 9). In India itself, there are at least 5.29 million people living with dementia currently, and this number is expected to double by 2035 (6). The prevalence of undetected dementia is also significantly high globally. It is estimated to be currently at 61.7%, with India and China having a higher proportion compared to Europe and the USA (10).

There are various barriers to the diagnosis of dementia in LMICs. Major factors include low awareness, inadequate healthcare resources, and scarcity of diagnostic tools that are culturally and linguistically valid (1, 8, 11). As a result, both under-detection and overdiagnosis of dementia are possible (11, 12). Hence, it is important that reliable diagnostic tools and instruments are developed that are culturally, educationally, and linguistically valid and can help in early and accurate diagnosis (12, 13). Additionally, the use of diagnostic tests that can be harmonized with future global studies is crucial. There have been some efforts toward developing comprehensive neuropsychological test batteries for use in various languages such as 10/66 global dementia studies (14), the Consortium to Establish a Registry for Alzheimer's Disease (CERAD) neuropsychological battery (15), the NIMHANS neuropsychological battery for the elderly (16), the Spanish English Neuropsychological Assessment Scales (SENAS) (17), and the international harmonization standards proposed by the National Institute of Neurological Disorders and Stroke (NINDS) and the Canadian Stroke Network (CSN) (18, 19). However, except for the studies by 10/66 dementia research groups, majority have been conducted in the developed world.

A significant amount of variance has been detected in the prevalence rate of dementia in India, a country with a large and diverse population (6, 7, 20–25). Variability in sociodemographic factors; genetic, protective, and risk factors; and methodological factors could account for these differences. To unravel the complex nature of dementia, research priorities to examine such large variations in dementia detection should be set, particularly in LMICs (26).

Variability in dementia-screening instruments and the use of different diagnostic methods and criteria contribute significantly to regional variability in reported dementia prevalence. To accurately establish the incidence or prevalence rates of dementia that are comparable across diverse populations, it is crucial that diagnostic instruments are harmonized by developing standardized procedures that are sensitive toward linguistic, educational, and cultural variability in populations (3). Additionally, efforts should also be made to increase the availability and ease of accessibility of these diagnostic instruments across the societies that are limited in resources. Another major barrier to effective management is a delay in early detection and treatment due to the scarcity of skilled professionals. Training more personnel on standardized diagnostic tools will also be necessary for effective management. These challenges exist not only for the diagnosis, treatment, and care of dementia but also for a majority of other mental health conditions (27).

To overcome these major barriers, a multidisciplinary group of neurologists, neuropsychologists, speech and language pathologists, and experts from related fields collaborated on a project funded by the Indian Council of Medical Research (ICMR) (http://icmr.nic.in). The efforts put forth by the group focused upon development, adaption, and validation of a comprehensive cognitive and functional test, the ICMR-Neurocognitive Toolbox (ICMR-NCTB) protocol, in five Indian languages (Hindi, Bengali, Telugu, Kannada, and Malayalam) with sensitivity toward different literacy levels across India (28). This test battery was developed to screen and diagnose dementia and mild cognitive impairment in the early stages, across the country (29), and to be suitable for conducting global collaborative research in cognitive disorders (28). The ICMR-NCTB has been validated for the diagnosis of MCI in the Indian context and demonstrated a good sensitivity of 81.1% and specificity of 88.8% to diagnose all-cause MCI (29). The usefulness of the ICMR-NCTB to diagnose dementia in the context of India requires to be established (30). In the background of a high burden of dementia due to Alzheimer's disease (AD) and vascular dementia (VaD) in India, the present study aimed to determine the validity of the ICMR-NCTB for the diagnosis of dementia in the context of linguistic heterogeneity in India.

## Methods

The ICMR established a collaboration between six academic institutions representing different linguistic states of India to develop and validate a cognitive test battery, to diagnose dementia in a standardized manner. Six centers that participated in this study are (1) Nizam's Institute of Medical Sciences (NIMS), Hyderabad, for Telugu and Hindi (the coordinating center); (2) All India Institute of Medical Sciences (AIIMS), Delhi, for Hindi; (3) Sri Chitra Tirunal Institute of Medical Science and Technology (SCTIMST), Trivandrum, for Malayalam; (4) National Institute of Mental Health and Neurosciences (NIMHANS) and Manipal Hospital, Bangalore, for Kannada; (5) Jawaharlal Nehru Medical College, Belgaum, for Kannada; and (6) Apollo Gleneagles, Kolkata, for Bengali. A multidisciplinary expert group collaborated toward the development of the ICMR Neurocognitive Toolbox (ICMR-NCTB) in five Indian languages (Hindi, Bengali, Telugu, Kannada, and Malayalam), to standardize the diagnosis of dementia in India (28).

The adaptation and validation involved a systematic process that included a review of existing international and national efforts at standardizing dementia diagnosis to identify culturally appropriate tests for the Indian context, adaptation for its use in five Indian languages and for both literates and illiterates, and validation in a cohort of individuals with normal cognition, mild cognitive impairment, and dementia across the multiple centers. This process has been detailed in an earlier report (28).

The ICMR-NCTB consisted of a range of tests that evaluate the major cognitive domains: (a) tests of cognition for the various domains of attention-executive functions: Trail Making Test A & B (TMT A & B) (31) and Category Fluency (32); memory: Verbal Learning Test—Total Learning and Delayed Recall (VLT—TL & DR) (33) and Modified Taylor Complex Figure Test—Delayed Recall (MTCF—DR) (34); and language (Picture Naming Test-PNT) and visuospatial skills (Modified Taylor Complex Figure test—Copy); and (b) questionnaires on behavior and functional activities: Geriatric Depression Scale (GDS) (35), Instrumental Activities of Daily Living—Elderly (IADL-E) (36), Neuropsychiatric Inventory (NPI) (37), Informant Questionnaire on Cognitive Decline in Elderly (IQCODE) (38), and RAND Short Form Health Survey (RAND SF-36) (39). A uniform protocol for the diagnosis of normal cognition and dementia due to neurodegenerative diseases was followed in all five centers (28).

Patients were recruited from out-patient services of neurology, geriatric, and internal medicine clinics of the participating hospitals, and healthy controls were randomly drawn from senior citizen centers, community outreach services, and healthy relatives of patients in the clinics. The detailed demographic, cognitive, and medical history of participants was collected to determine the eligibility for participation. Participants who fulfilled the following inclusion criteria for healthy controls were recruited: individuals >40 years and consented to participate; individuals with no history of head injury, infections, stroke, and other neurological, systemic, medical, or psychiatric disorders that can cause cognitive impairment; and those with no significant hearing or visual impairments that could interfere with the testing. A standard and harmonized case record form was used to collect sociodemographic information and neurocognitive and functional data.

An experienced cognitive neurologist evaluated all subjects, and experienced psychologists administered the gold standard tests on all the participants. Participants without any subjective cognitive complaints and scored normally on Addenbrooke's Cognitive Examination-III (ACE-III), Clinical Dementia Rating (CDR), Rey Auditory Verbal Learning Test (RAVLT), and Color Trails Test (CTT) were considered as healthy controls (28, 29). Participants with dementia were diagnosed based on clinical evaluation and the presence of impaired cognitive functions, as indicated by their scores on ACE-III (40) and CDR (28, 41). The dementia diagnosis was done based on the standard DSM-IV criteria (42). Patients were further classified into dementia subtypes: AD was diagnosed in patients who fulfilled the NIA-AA criteria for probable and possible AD (43), vascular dementia was diagnosed in patients who fulfilled the NINDS-AIREN criteria (44), and FTD was diagnosed based on the criteria by Rascovsky et al. (45). Persons diagnosed to have MCI were excluded from this study. The diagnosis of MCI was made based on the modified Petersen criteria (46). The recruited participants were subsequently administered with the complete ICMR-NCTB by a team of psychologists and clinicians who were blind to the diagnosis. The research ethics committee of all the participating centers approved the study, and consent was obtained from all the participants and their family caregivers.

### Statistical Analysis

To compare the demographic data and neuropsychological test scores of patients with dementia and controls, an independent sample *t*-test for normally distributed continuous data or Mann—Whitney *U*-test for non-normal data, χ2 tests or Fisher's exact tests for categorical data, and trend test for ordinal data were used as appropriate. The test scores were represented in mean and standard deviation except TMT A & B scores, which is represented in median and interquartile range due to variability in the scores in the dementia group. The external validity of the battery was determined by the receiver operating curve (ROC) using the area under the curve (AUC). The optimum cutoff scores were established with corresponding sensitivity and specificity levels. All statistical analyses were performed using SPSS Statistics for Windows, version 23.0.

## Results

A total of 1,141 participants were recruited that included 991 controls and 185 patients with dementia. After matching the groups for age, education, and gender, 354 participants (222 controls and 132 patients with dementia) were included for further analysis. The patients were diagnosed as Alzheimer's disease (AD), vascular dementia (VaD), and frontotemporal Dementia (FTD): AD−65, VaD−45, and FTD−22. The mean age of the healthy controls and patients with dementia was 65 years and 66 years, respectively. Participants were from both urban and rural backgrounds: 71% were controls and 77% of patients were urban dwellers. Out of 132 patients with dementia, 61 (46.30%) reported to have very mild dementia, 47 (35.60%) mild dementia, 18 (13.60%) moderate, and 6 (4.50%) severe on the Clinical Dementia Rating Scale (CDR). Because of the heterogeneity in demographic characteristics in the overall cohort, language-wise analysis was conducted (Hindi: controls−40, dementia−20; Bengali: controls−45, dementia−29; Telugu: controls−45, dementia−33; Kannada: controls−57, dementia−15; and Malayalam: controls−35, dementia−35). The demographic characteristics and cognitive test scores on ACE-III of healthy controls and patients with dementia are presented in Table 1. Both healthy controls and dementia patient groups were matched for age, education, and gender in all the language groups. Healthy controls performed significantly better on ACE-III than patients with dementia [*t*_(330)_ = 18.87, *p* < 0.001) in all the five language groups.

**Table 1:** Demographic profile of Hindi, Bengali, Telugu, Kannada, and Malayalam speaking healthy controls and dementia.

**Language and diagnosis**	**N**	**Age (years)**	**Gender**	**Years of education**	**ACE-III**
		**mean (SD)**	**(male, female) %**	**mean (SD)**	**mean (SD)**
Hindi—controls	40	57.10 (6.25)	67.5, 32.5	14.03(3.40)	86.37 (7.53)
Hindi—dementia	20	61.00 (9.06)	55.0, 45.0	13.15(2.98)	56.33 (19.45)
Bengali—controls	45	66.27 (6.18)	71.1, 28.9	11.62(4.28)	88.62 (7.24)
Bengali—dementia	29	67.03 (10.47)	65.5, 34.5	12.21(3.74)	57.69 (16.11)
Telugu—controls	45	66.29 (3.93)	66.7, 33.3	14.20(4.14)	93.62 (4.13)
Telugu—dementia	33	65.55 (8.56)	54.5, 45.5	13.03(5.32)	70.52 (19.02)
Kannada—controls	57	64.47 (3.10)	40.4, 59.6	12.00(3.49)	87.77 (7.59)
Kannada—dementia	15	67.20 (9.11)	46.7, 53.3	11.67(4.25)	37.20 (21.51)
Malayalam—controls	35	68.91 (5.39)	65.7, 34.3	12.71(2.56)	92.09 (4.08)
Malayalam—dementia	35	70.11 (5.91)	77.1, 22.9	11.50(2.59)	66.43 (13.15)

*SD, standard deviation; ACE-III, Addenbrooke's Cognitive Examination*.

*Missing values: ACE-III (controls, patients)—Hindi (2, 5), Bengali (8, 0), Telugu (6, 0), Kannada (0, 0), Malayalam (1, 0)*.

A significant difference in the mean (median) scores between healthy controls and patients with dementia was observed on all the tests of ICMR-NCTB (Table 2). Dementia patients took more time on TMT A & B and scored lower on category fluency than healthy controls in five Indian languages, which is indicative of significant impairment in their attention and executive functioning (*TMT A*—Hindi: *U* = 187, *n* = 45 *p* = 0.194; Bengali: *U* = 185, *n* = 72, *p* < 0.001; Telugu: *U* = 149.50, *n* = 65, *p* < 0.001; Kannada: *U* = 127.50, *n* = 47, *p* < 0.001; Malayalam: *U* = 166, *n* = 66, *p* < 0.001), *TMT B*—Hindi: *U* = 235, *n* = 45 *p* = 0.862; Bengali: *U* = 577, *n* = 70, *p* = 0.832; Telugu: *U* = 214.40, *n* = 61, *p* = 0.003; Kannada: U = 90.50, *n* = 43, *p* = 0.482; Malayalam: *U* = 143.50, *n* = 65, *p* < 0.001), and *category fluency*—Hindi: *t*_(55)_ = 4.46, *p* < 0.001; Bengali: *t*_(67)_ = 5.32, *p* < 0.001, Telugu: *t*_(74)_ = 6.61, *p* < 0.001; Kannada: *t*_(70)_ = 9.15, *p* < 0.001; Malayalam: *t*_(68)_ = 9.09, *p* < 0.001). Similarly, patients with dementia performed poorly on PNT, VLT (total learning and delayed recall), and MTCF copy and delayed recall compared to healthy controls, suggesting difficulties in language, memory, and visuospatial abilities (*PNT*—Hindi: *t*_(53)_ = 9.49, *p* < 0.001; Bengali: *t*_(55)_ = 6.07, *p* < 0.001; Telugu: *t*_(56)_ = 4.85, *p* < 0.001; Kannada: *t*_(53)_ = 12.05, *p* < 0.001; Malayalam: *t*_(68)_ = 6.68, *p* < 0.001), *VLT (total learning and delayed recall)*—Hindi: *t*_(55)_ = 6.16, *p* < 0.001; Bengali: *t*_(70)_ = 5.42, *p* < 0.001, Telugu: *t*_(74)_ = 4.68, *p* < 0.001; Kannada: *t*_(70)_ = 4.64, *p* < 0.001; Malayalam: *t*_(68)_ = 7.69, *p* < 0.001), and *MTCF copy and delayed recall* (Hindi: *t*_(45)_ = 5.04, *p* < 0.001; Bengali: *t*_(51)_ = 10.29, *p* < 0.001, Telugu: *t*_(76)_ = 5.23, *p* = 0.024; Kannada: *t*_(39)_ = 5.46, *p* < 0.001; Malayalam: *t*_(61)_ = 10.99, *p* < 0.001).

**Table 2:** ICMR-NCTB test scores of healthy controls and patients with dementia in Hindi, Bengali, Telugu, Kannada, and Malayalam.

**Language and diagnosis**	**Attention and executive functions**			**Episodic memory**			**Language**	**Visuospatial functions**
	**Trail Making Test (TMT) A in seconds Median [IQR]**	**Trail Making Test (TMT) B in secondsMedian [IQR]**	**Category Fluency (animals) Mean (SD)**	**Verbal Learning Test Delayed RecallMean (SD)**	**Verbal Learning Test Total Learning Mean (SD)**	**Modified Taylor Complex Figure Test (MTCF)—Delayed RecallMean (SD)**	**Picture Naming Test (PNT) Mean (SD)**	**Modified Taylor Complex Figure Test (MTCF)- CopyMean (SD)**
Hindi—controls	68.00 [41.00]	162.00 [62.00]	9.53 (2.75)	5.05 (1.93)	17.21 (4.06)	15.42 (6.74)	88.51 (4.03)	34.88 (1.82)
Hindi—dementia	89.50 [121.00]	180.00 [300.00]	5.58 (3.85)	1.11 (1.66)	8.68 (6.33)	4.70 (6.95)	49.31 (25.35)	19.57 (13.46)
Bengali—controls	71.00 [41.00]	203.00 [139.00]	15.65 (4.02)	4.72 (1.93)	19.49 (4.56)	15.39 (5.38)	81.55 (7.28)	33.85 (2.19)
Bengali—dementia	151.00 [83.00]	300.00 [300.00]	9.90 (4.95)	1.55 (1.88)	13.24 (5.12)	2.45 (3.46)	65.07 (14.53)	12.86 (12.77)
Telugu—controls	60.51[25.00]	160.00[77.00]	14.38 (3.73)	5.35 (2.14)	18.85 (3.86)	18.61 (6.78)	86.07 (3.94)	34.95 (1.71)
Telugu—dementia	90.5[38.8]	200.00[93.00]	8.75 (3.53)	2.65 (2.84)	8.39 (8.24)	9.33 (8.84)	63.80 (27.03)	33.97 (38.16)
Kannada—controls	70.00 [31.00]	157.00 [91.00]	12.11 (4.09)	4.23 (1.91)	17.02 (2.48)	20.03 (8.06)	83.80 (5.18)	34.69 (1.89)
Kannada—dementia	359.00 [240.5]	618.00 [310.00]	2.20 (1.61)	1.66 (1.87)	7.33 (5.12)	2.71(4.79)	35.07 (24.53)	9.14 (11.55)
Malayalam—controls	79.00 [39.00]	205.00 [69.00]	14.43 (2.27)	4.60 (1.79)	18.09 (3.00)	17.40 (6.59)	79.31 (5.89)	35.16 (1.55)
Malayalam—dementia	175.00 [154.00]	413.00 [288.00]	8.11 (3.43)	0.97 (1.93)	11.26 (4.31)	2.74 (3.72)	61.68 (13.93)	20.15 (13.74)

*IQR, interquartile range; SD, standard deviation*.

*Missing values*.

*Hindi: TMT A and B: controls: 13, patients: 10; category fluency (animals): controls: 1, patients: 1; VLT: controls: 0, patients: 4; PNT: controls: 2, patients: 3; MTCF: controls: 5, patients: 9*.

*Bengali: TMT A and B: controls: 4, patients: 12; category fluency (animals): controls: 5, patients: 0; VLT: controls: 2, patients: 0; PNT: controls: 7, patients: 0; MTCF: controls: 21, patients: 0*.

*Telugu: TMT A and B: controls: 14, patients: 12; category fluency (animals): controls: 1, patients: 1; VLT: controls: 0, patients: 2; PNT: controls: 15, patients: 5; MTCF: controls: 5, patients: 9*.

*Kannada: TMT A and B: controls: 19, patients: 1; PNT: controls: 17, patients: 15; MTCF: controls: 21, patients: 8*.

*Malayalam: TMT A and B: Controls: 4, Patients: 4; MTCF: Controls: 4, Patients: 2*.

**Figure 1:**
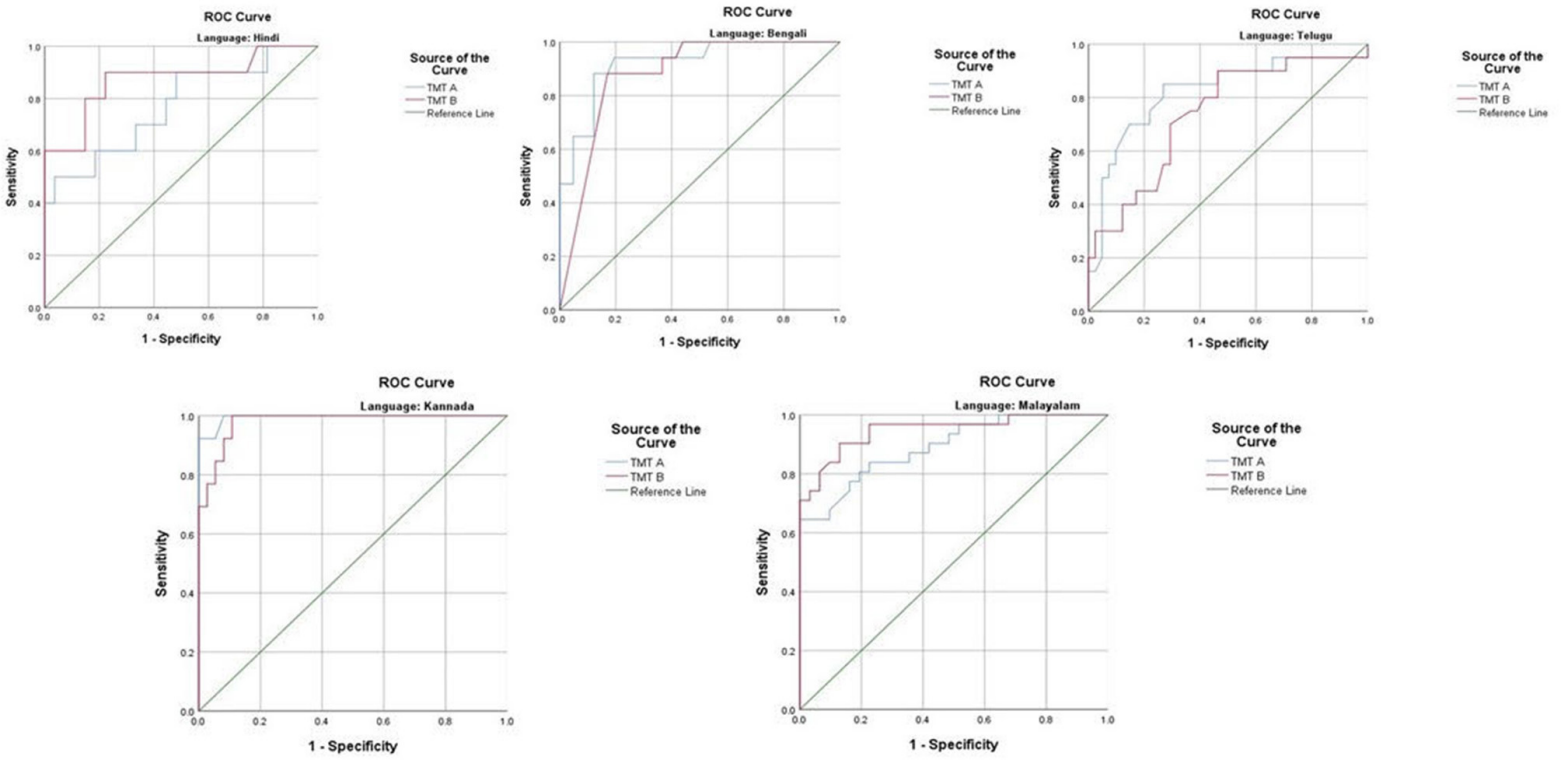
Receiver operating curve (ROC) of Trail Making Test (A and B) in diagnosing dementia across five languages.

**Figure 2:**
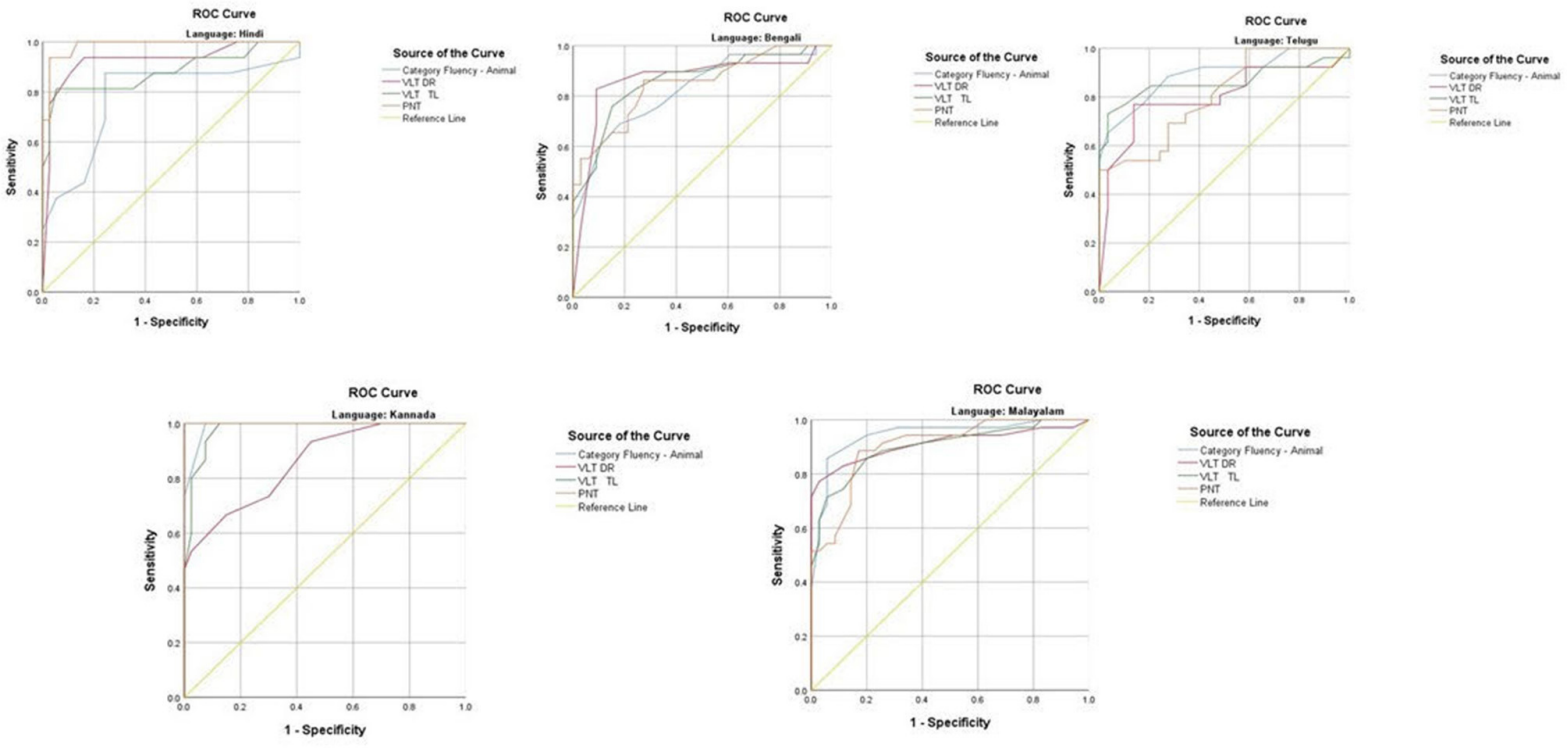
Receiver operating curve (ROC) of Category Fluency-Animal, Verbal Learning Test: Delayed Recall and Total Learning (VLT DR and VLT TL), and Picture Naming Test (PNT) in diagnosing dementia across five languages.

The ROC revealed that the majority of the ICMR-NCTB tests had good discriminating power in differentiating cognitively impaired participants from the normal healthy group across five languages (see Table 3) (*TMT A*: AUC: 0.79-0.99, CI: [0.69, 1.00]; *TMT B*: AUC: 0.74-0.98, CI: [0.60, 1.00]; *Category Fluency Animal*: AUC: 0.77-0.99, CI: [0.59, 1.00]; *Verbal Learning Test Delayed Recall:* AUC: 0.79-0.94, CI: [0.67, 0.99]; *Verbal Learning Test Total Recall:* AUC: 0.89-0.98, CI: [0.76, 1.00]; and *Picture Naming Test*: AUC: 0.81-1.00, CI: [0.69, 1.00]). The ROC analysis for the MTCF test could not be done due to small sample size, as individuals with <7 years of education and severe cases of dementia were not able to perform the task. The tests of ICMR-NCTB showed high sensitivity and the specificity at optimal cutoff scores, suggesting the ability of the tests to diagnose dementia in five Indian languages, namely, Hindi, Bengali, Telugu, Kannada, and Malayalam (Table 4; Figures 1, 2).

**Table 3:** Area under curves of ICMR-NCTB tests across five languages.

**Test**	**Hindi**	**Bengali**	**Telugu**	**Kannada**	**Malayalam**
**Attention and executive functions**					
Trail MakingTest (TMT) A(seconds)	AUC = 0.79CI: [0.69, 0.98]	AUC = 0.92CI: [0.85, 0.99]	AUC = 0.82CI: [0.69, 0.94]	AUC = 0.99CI: [0.98, 1.00]	AUC = 0.89 CI: [0.81, 0.97]
Trail MakingTest (TMT) B (seconds)	AUC = 0.87CI: [0.72, 1.00]	AUC = 0.88CI: [0.79, 0.97]	AUC = 0.74CI: [0.60, 0.87]	AUC = 0.98CI: [0.95, 1.00]	AUC = 0.95 CI: [0.89, 1.00]
Category fluency(animals)	AUC = 0.77CI: [0.58, 0.95]	AUC = 0.86CI: [0.76, 0.97]	AUC = 0.89CI: [0.81, 0.98]	AUC = 0.99CI: [0.97, 1.00]	AUC = 0.93 CI: [0.87, 0.99]
**Episodic memory**					
Verbal LearningTest DelayedRecall	AUC = 0.92CI: [0.81, 1.00]	AUC = 0.87CI: [0.76, 0.98]	AUC = 0.79CI: [0.67, 0.93]	AUC = 0.83CI: [0.72, 0.91]	AUC = 0.94 CI: [0.88, 0.99]
Verbal LearningTest Total Recall	AUC = 0.90CI: [0.76, 1.00]	AUC = 0.89CI: [0.81, 0.98]	AUC = 0.86CI: [0.75, 0.97]	AUC = 0.98CI: [0.95, 1.00]	AUC = 0.90 CI: [0.83, 0.98]
**Language**					
Picture NamingTest (PNT)	AUC = 0.98CI: [0.95, 1.00]	AUC = 0.92CI: [0.82, 0.99]	AUC = 0.81CI: [0.69, 0.92]	AUC = 1.00CI: [1.00, 1.00]	AUC = 0.93 CI: [0.87, 0.99]

*AUC, area under curve; CI, confidence interval*.

**Table 4:** Optimal cutoff scores and the respective sensitivity and specificity of ICMR-NCTB tests for diagnosis of dementia across five languages.

**Test**	**Hindi**	**Bengali**	**Telugu**	**Kannada**	**Malayalam**
**Attention and executive functions**					
Trail Making Test (TMT) A(seconds)	Cutoff: > 95 Sensitivity: 71.43 Specificity: 81.48	Cutoff: > 105 Sensitivity: 92.30 Specificity: 88.40	Cutoff: > 74 Sensitivity: 75.00 Specificity: 85.40	Cutoff: > 144 Sensitivity: 92.90 Specificity: 100.00	Cutoff: > 115 Sensitivity: 75.76 Specificity: 83.87
Trail Making Test (TMT) B(seconds)	Cutoff: > 204 Sensitivity: 90.00 Specificity: 77.80	Cutoff: > 298 Sensitivity: 88.20 Specificity: 82.90	Cutoff: > 180 Sensitivity: 70.00 Specificity: 70.70	Cutoff: > 267 Sensitivity: 100.00 Specificity: 89.20	Cutoff: > 290 Sensitivity: 90.30 Specificity: 87.10
Category Fluency (animals)	Cutoff: ≤ 8 Sensitivity: 89.50 Specificity: 73.70	Cutoff: ≤ 11 Sensitivity: 65.52 Specificity: 82.50	Cutoff: ≤ 8 Sensitivity: 100.00 Specificity: 97.50	Cutoff: ≤ 8 Sensitivity: 100.00 Specificity: 80.70	Cutoff: ≤ 11 Sensitivity: 85.70 Specificity: 94.30
**Episodic memory**					
Verbal Learning Test Delayed Recall	Cutoff: ≤ 3 Sensitivity: 89.50 Specificity: 84.20	Cutoff: ≤ 2 Sensitivity: 82.80 Specificity: 90.70	Cutoff: ≤ 3 Sensitivity: 71.00 Specificity: 84.40	Cutoff: ≤ 2 Sensitivity: 73.30 Specificity: 82.50	Cutoff: ≤ 2 Sensitivity: 82.86 Specificity: 88.57
Verbal Learning Test Total Recall	Cutoff: ≤ 11 Sensitivity: 78.90 Specificity: 94.70	Cutoff: ≤ 16 Sensitivity: 75.80 Specificity: 83.70	Cutoff: ≤ 16 Sensitivity: 87.10 Specificity: 80.00	Cutoff: ≤ 14 Sensitivity: 100.00 Specificity: 87.70	Cutoff: ≤ 13 Sensitivity: 71.40 Specificity: 94.30
**Language**					
Picture Naming Test (PNT)	Cutoff: ≤ 81 Sensitivity: 93.70 Specificity: 97.40	Cutoff: ≤ 77 Sensitivity: 86.20 Specificity: 73.70	Cutoff: ≤ 73 Sensitivity: 71.40 Specificity: 73.30	Cutoff: ≤ 69 Sensitivity: 100.00 Specificity: 100.00	Cutoff: ≤ 74 Sensitivity: 88.60 Specificity: 82.90

## Discussion

In the present study, we assessed the diagnostic accuracy of the tests included in the ICMR Neurocognitive Toolbox in detection of dementia, across five Indian languages (Hindi, Bengali, Telugu, Kannada, and Malayalam). The ICMR-NCTB in all the five Indian languages met standardized test requirements, which indicates that the test adaption and standardization were successful across languages. This study confirms the utility of majority of the tests included in the ICMR-NCTB as effective instruments for the diagnosis of dementia, particularly with a relatively high sensitivity and specificity in a linguistically diverse context. Overall, the ICMR-NCTB appears promising in terms of validity based upon standard criteria for evaluating a dementia diagnostic test in the Indian context (28, 47).

Dementia is one of the most important independent contributors to disability in elderly especially in low- to middle-income countries (LMICs) where the resources to diagnose and manage dementia are limited. While specialized services for dementia are increasingly available in high-income countries, such facilities are lacking in LMICs. In addition, primary care physicians in developing countries do not receive suitable training to diagnose dementia and its subtypes. The gap in the diagnosis of dementia is further widened by the cross-cultural differences in understanding dementia due to linguistic and educational diversity. Therefore, a valid test battery that can be applied by clinicians and neuropsychologists in diagnosing dementia is crucial in the linguistically and educationally diverse Indian context.

The development and validation of a comprehensive NCTB protocol for the diagnosis of dementia, harmonized in five different languages, was an important facet of this study. It was established by following a common methodology that was applied on a large cohort consisting of persons with diverse linguistic profiles, which enabled it to be effectively utilized to detect cognitive deficits in early stages of dementia and help in reducing the variability in clinical diagnosis in hospitals and clinics across India. The main finding in our study was that the tests included in the ICMR-NCTB were found to be sensitive and specific in the identification of dementia in LMICs in all of the five Indian languages.

The external validity of each individual test included in the ICMR-NCTB was determined by the receiver operating curve (ROC) using the area under curve (AUC), and optimum cutoff scores were established with corresponding sensitivity and specificity levels.

Our study showed that the Trail Making Test-A, a test of attention, included in the ICMR-NCTB accurately differentiated patients with dementia from healthy control participants with high sensitivity ranging from 71 to 93% and specificity ranging from 81 to 100% at the optimal cutoff points ranging from (>) 74 to 144 across the five Indian languages. Similarly, the Trail Making Test-B, a test of executive function, also accurately differentiated the patient group from healthy individuals with sensitivity and specificity ranging from 70 to 100% and 71to 89%, respectively, at optimal cutoff points ranging from (>) 180 to 298 across languages. Few moderate and severe patients with dementia were not able to complete the Trail Making Test due to the typical decline in attention and executive functions that are evident in the later stages of the disease (48).

Category fluency (animals) showed high sensitivity (86-100%) and specificity (74-98%) at optimal cutoff points ranging from 8 to 11, except in Bengali where the sensitivity of the category fluency task was moderate (66%) with good specificity (83%) at an optimal cutoff point of 11. This finding is in agreement with the verbal fluency test included in the Consortium to Establish a Registry for Alzheimer's Disease (CERAD) neuropsychological battery which showed higher sensitivity (75%) and specificity (74%) at an optimal cutoff point of <17 for the category fluency task (15). The lower category fluency cutoff score in the ICMR-NCTB compared to CERAD neuropsychological battery might be due to the inclusion of moderate and severe dementia patients. The NIMHANS Neuropsychological Battery for the Elderly demonstrated good discriminability for the animal fluency task (AUC = 0.99, 95% CI [0.96, 0.99]) (16) that was comparable to the discriminability findings of category fluency (AUC = 0.77-0.99, 95% CI [0.58, 1.00]) findings of our study.

The episodic memory tests of the ICMR-NCTB (verbal learning test—TL and verbal learning test—DR) accurately differentiated patients with dementia from the healthy control group, which is consistent with the criteria to diagnose majority of dementia subtypes including AD (49) that highlight episodic memory impairment in patients with dementia. The verbal learning test-DR showed high sensitivity and specificity ranging from 71 to 90% and 83 to 91%, respectively, with optimal cutoff points ranging from 2 to 3, and the verbal learning test-TL showed a sensitivity of 71-100% and specificity of 80-95% at optimal cutoff points ranging from 11 to 16. The high discriminability of the episodic memory tests of ICMR-NCTB compares well with the word list DR (sensitivity = 94%, specificity = 85%, cutoff = 5) and word list learning (sensitivity = 90%, specificity = 83%, cutoff = 17) of CERAD neuropsychological battery (15). The cutoff scores of verbal learning test are consistent with studies done in LMICs like Brazil. A Brazilian epidemiological study derived a cutoff score of 3 in the literate group and 1 in the illiterate group for delayed recall of a word list test from the CERAD neuropsychological battery (50). A different study from India also established a cutoff score of 3 for the delayed verbal memory test (33), from Kolkata cognitive screening battery, which is also consistent with our study. The word list-delayed recall (AUC = 0.99; 95% CI [0.97, 0.99]) of NIMHANS neuropsychological battery for the elderly revealed the highest discriminability (16), which is comparable with the verbal learning test DR (AUC = 0.79-0.94; 95% CI [0.67, 0.99]).

The Picture Naming Test included in the ICMR-NCTB showed high sensitivity (71-100%) and specificity (73-100%) at optimal cutoff points ranging from 69 to 81 (maximum score = 90), which compares favorably well with the naming test of CERAD neuropsychological battery with a sensitivity of 68% and specificity of 76% at an optimal cutoff point of 12 (maximum score = 15) (15).

The high sensitivity and specificity of the majority of the tests included in the ICMR-NCTB for diagnosing dementia favorably compares to that of other cognitive test batteries such as the Spanish and English Neuropsychological Assessment Scales (SENAS) (51) battery with 80% sensitivity and specificity for a combination of word list learning and object naming to diagnose dementia. The sensitivity of the diagnostic algorithm against clinically diagnosed dementia in the widely used 10/66 pilot samples was 94%, and the specificity was 97% in people with high education and 93% in individuals with low education (52), which is comparable with the sensitivity and specificity of the ICMR-NCTB tests.

Tests included in the NINDS-CSN battery include Animal Naming Test (ANT), Wechsler Adult Intelligence Scale (WAIS) digit symbol coding, Trail Making Test A & B, Boston Naming Test (BNT), Rey—Osterrieth Complex Figure Test (RCFT) copy, Verbal Learning Test-delayed recall, and RCFT-delayed recall (53), which is very similar to the range of tests included in the ICMR-NCTB. Z-scores were derived, and the external validity evaluated by AUC for the 60-min protocol of the NINDS-CSN battery was 0.88 [95% (CI), 0.81, 0.95]. Although a direct comparison of ICMR-NCTB with NINDS-CSN battery cannot be made, the AUC of ICMR-NCTB tests ranged from 0.73 to 1.00, which indicates a good discriminating power in diagnosing dementia, similar to NINDS-CSN battery.

An important feature of the study is that it is unique in comprehensively addressing the validity of each neuropsychological test included in the ICMR toolbox in a linguistically, educationally, and culturally heterogeneous population. A further strength of the ICMR-NCTB is that tests for all major cognitive domains of attention/executive function, language, memory, and visuospatial functions are incorporated and optimum cutoff points with corresponding sensitivity and specificity of various cognitive domains in five Indian languages are provided separately. This is crucial for the diagnosis of dementia subtypes: AD, VaD, and FTD that have characteristic cognitive profiles. While AD is a disorder of memory especially in the early stages (54), VaD is characterized by prominent executive dysfunction (55) and frontotemporal dementia syndromes present with language and/or executive function impairment (56). The advantage of inclusion of tests of all major cognitive domains in the ICMR-NCTB is reflected in the relatively high diagnostic sensitivity and specificity of majority of the cognitive domains in this dementia cohort consisting of multiple subtypes. While the study has established successful discriminability between dementia and controls across all tests, the most efficacious combination of measures discriminating healthy controls from patients with dementia is yet to be determined.

There were certain limitations identified. (i) The study was conducted in a literate population, and patients with dementia studied were relatively young. (ii) We had a relatively small sample in the Kannada dementia group which might be one of the reasons for the high sensitivity and specificity of ICMR-NCTB tests in Kannada. (iii) Differing proportions of dementia subtypes in our dementia cohort might have led to the differences in ages across dementia patients in five language groups. (iv) We did not have enough numbers for establishing diagnostic validity separately for subtypes of dementia. (v) For clinical and research generalizability, the test battery will need to be adapted to the illiterate group and in larger numbers in the future. (vi) The findings of the current study are applicable to dementia cohorts seen in memory clinics and specialized centers only as the study was conducted in academic medical centers. (vii) There was a variation in the cutoff scores across languages for the Modified Taylor Complex Figure test (MTCF) copy and delayed recall tests, as the sample size was not adequate due to the inability of the low-educated participants and advanced dementia patients to perform the test. Therefore, the MTCF test could not be validated in the current study and the tool might not be applicable for the low literate population in the Indian context. This is planned during the next phase of the study, in larger and more diverse clinical and community populations to further validate the ICMR-NCTB.

To conclude, we were successfully able to validate a cognitive test battery in five different languages that is harmonized culturally and linguistically to diagnose dementia in India. The high specificity and sensitivity of the tests included in the ICMR-NCTB highlight its ability to detect dementia across languages. Our study thus establishes a benchmark for dementia research in India and will prove to be an invaluable tool for clinical practice and for multicentric preventive and therapeutic research in a socio-linguistically diverse context.

## Data Availability Statement

The raw data supporting the conclusions of this article will be made available upon request, without undue reservation. Requests to access the datasets should be directed to the corresponding author.

## Ethics Statement

The studies involving human participants were reviewed and approved by Research Ethics Committee of Nizam's Institute of Medical Sciences, Hyderabad; All India Institute of Medical Sciences Ethics Committee, Delhi; Institutional Ethics Committee, Apollo Gleneagles Hospital, Kolkata; Ethics Committee of Manipal Hospital, Bengaluru; and Institutional Ethics Committee, Sree Chitra Tirunal Institute for Medical Sciences and Technology, Kerala. The patients/participants provided their written informed consent to participate in this study.

## Author Contributions

MV contributed toward project implementation, data collection, analyses, and manuscript writing. MT, AN, and SA contributed toward tool development and adaptation, project implementation, data collection, analyses, and manuscript writing. APap contributed data collection, data analyses, and manuscript writing. FV contributed toward statistical and data analyses. RD, MS, AS, AG, RNM, GI, RM, JN, SR, PS, and RV contributed toward tool development and adaptation, project implementation, data collection, analyses, and manuscript editing. APau, MP, US, and SK are expert panelists who contributed toward tool development and adaptation, project implementation, and manuscript editing. FA, GD, TM, SM, RH, JS, RK, AK, RN, LS, and YV contributed toward project implementation, data collection, and manuscript editing. All authors contributed to the article and approved the submitted version.

## Funding

This work was supported by the Indian Council for Medical Research under Grant [SWG/Neuro/32/2017-NCD-1].

## Conflict of Interest

The authors declare that the research was conducted in the absence of any commercial or financial relationships that could be construed as a potential conflict of interest.

## Publisher's Note

All claims expressed in this article are solely those of the authors and do not necessarily represent those of their affiliated organizations, or those of the publisher, the editors and the reviewers. Any product that may be evaluated in this article, or claim that may be made by its manufacturer, is not guaranteed or endorsed by the publisher.
